# Cerebrovascular Pressure Reactivity Measures: Index Comparison and Clinical Outcome in Patients With Traumatic Brain Injury Treated According to an Intracranial Pressure–Focused Management: Rejection of the Null Hypothesis

**DOI:** 10.1089/neur.2023.0074

**Published:** 2023-12-26

**Authors:** Axel Risinger Liljegren, Camilla Brorsson, Marcus Karlsson, Lars-Owe D. Koskinen, Nina Sundström

**Affiliations:** ^1^ Department of Clinical Science-Neurosciences, Radiation Physics, Biomedical Engineering, Umeå University, Umeå, Sweden.; ^2^ Department of Surgery and Perioperative Sciences, Radiation Physics, Biomedical Engineering, Umeå University, Umeå, Sweden.; ^3^ Department of Radiation Sciences, Radiation Physics, Biomedical Engineering, Umeå University, Umeå, Sweden.

**Keywords:** neurointensive care, neurosurgery, pressure reactivity indices, traumatic brain injury

## Abstract

The aim was to investigate whether the pressure reactivity indices PRx, long-PRx (L-PRx), and pressure reactivity (PR) are interchangeable as measures of vascular reactivity, and whether they correlate with clinical outcome when an intracranial pressure (ICP)-targeted treatment regimen is applied in patients with traumatic brain injury (TBI). Patients with TBI (*n* = 29) that arrived at the hospital within 24 h of injury were included. PRx and L-PRx were derived from Pearson correlations between mean arterial pressure (MAP) and ICP over a short- and long-time interval. PR was the regression coefficient between the hourly mean values of ICP and MAP. Indices were compared to each other, parameters at admission, and outcome assessed by the extended Glasgow Outcome Scale-Extended (GOSE) at 6 and 12 months. PRx and L-PRx had the strongest correlation with each other (*R* = 0.536, *p* < 0.01). A correlation was also noted between L-PRx and PR (*R* = 0.475, *p* < 0.01), but not between PRx and PR. A correlation was found between age and PRx (*R* = 0.482, *p* = 0.01). No association with outcome for any of the indices was found. PRx/L-PRx and L-PRx/PR were moderately correlated with each other. Age was associated with PRx. None of the indices correlated with outcome when our ICP treatment regime was applied. Part of our null hypothesis, that the three indices are associated with outcome, must be rejected. There was, however, an association between some of the indices. To further understand the relation of treatment regimes and pressure reactivity indices, a larger, randomized study is warranted.

## Introduction

Autoregulation is the brain's ability to respond to changes in arterial blood pressure (ABP) with either vasoconstriction or dilation, in an attempt to keep a constant cerebral blood flow (CBF) despite changes in cerebral perfusion pressure (CPP).^1^ The brain's autoregulatory ability can be eliminated globally or locally in case of trauma or injury.^2^ Thus, CBF may vary passively to blood pressure, which may increase the brain's susceptibility to changes in mean arterial blood pressure (MAP).^3^ Cerebral autoregulation (CA), expressed as different pressure reactivity (PR) indices, has in recent years gained some traction as a possible feature that can be monitored and used in individualizing management after traumatic brain injury (TBI) in both children and adults.^4–6^ There are no direct methods for monitoring autoregulation in humans, and most surrogate methods are based on relationships between intracranial pressure (ICP) and MAP.^7–9^ It is anticipated that with an intact autoregulating ability, a decrease in ABP will lead to arterial dilation, which keeps the CBF constant but with an increased cerebral blood volume and subsequently increased ICP, and vice versa. Thus, if ICP and ABP vary in the same direction, autoregulation is said to be poor.

The most used surrogate measure for monitoring CA is the pressure reactivity index (PRx) calculated as a correlation between ICP and MAP.^8^ PRx has been shown to correlate to neurological outcome,^2,10^ but requires high-frequency data (at least one sample per second), which is not standard in most neurosurgical centers or intensive care units (ICUs) in the world. Therefore, other methods have been developed, such as long PRx (L-PRx) and the PR method, where only minute values are necessary.^4,11^ L-PRx is based on similar correlations as PRx, whereas PR is based on the regression coefficient between ICP/MAP.^4^ There is no direct comparison between all of these methods, but the relationship between PRx and L-PRx has previously been explored.^12,13^ If the simpler indices L-PR and/or PR can provide similar information as PRx, many clinics that lack the opportunity for advanced data collection will be able to determine how the vascular reactivity of the brain, as a measure of CA, relates to the given treatment and clinical outcome when treating different types of injuries.

The CPP-oriented concept based on the Brain Trauma Foundation (BTF) guidelines is widely used.^14^ Correlations have been found between PRx, L-PRx, and PR and the outcome in groups treated with a CPP-oriented therapy.^11,15^ At Umeå University Hospital, an ICP-oriented approach, based on the Lund concept,^16^ is used that intends to keep ICP below 20 mm Hg while secondly allowing CPP down to 50 mm Hg in certain situations in order to avoid the negative effects of vasopressors and hypervolemia. No attempt is usually made to increase CPP above 60 mm Hg. In the latest BTF guidelines, a CPP is recommended that is closer to the Lund concept.^17–19^

In a recent European study, Collaborative European NeuroTrauma Effectiveness Research in TBI (CENTER-TBI),^20^ PRx was shown to relate to clinical outcome.^10,21^ However, the results have not been related to treatment methods. Given that at Umeå University Hospital only the ICP-oriented treatment concept is used, in this study, for the first time, the different indices described can be evaluated on patients solely managed according to this concept.

The purpose of this study was to compare PRx, L-PRx, and PR and investigate whether they are interchangeable as measures of cerebrovascular pressure reactivity in patients with TBI treated with an ICP-oriented therapy. It was also to investigate whether PRx, L-PRx, or PR are related to clinical outcome when an ICP-targeted treatment regimen is applied. Our null hypothesis, based on previous reported results, is that there is an association between the indices and clinical outcome as well as between the surrogate indices for CA.

## Methods

### Patient population

The patient cohort consisted of 29 patients treated in the ICU at Umeå University Hospital. All were prospectively recruited from January 2015 to December 2017. Patients of all ages with a clinical diagnosis of severe TBI, an indication for computed tomography that presented to the hospital within 24 h of injury, and where informed consent was obtained according to local and national requirements were included.^22^ Exclusion criteria were severe pre-existing neurological disorders.^22^ Data were not excluded after active cerebrospinal fluid drainage, performance of decompressive craniotomy, or pentobarbital treatment. Thus, our study reflects a real clinical situation as faced by the clinician.

### Ethics

The collected data were anonymized, and all procedures performed in this study were in accordance with the Regional Ethical Review Board (2014/1473-31/4, 2011-256-31M, 00-175, and 2013-43-31M) and with the 1964 World Medical Association Declaration of Helsinki and its later amendments.

### Data collection

All patient data were collected as a part of an ongoing local study and the CENTER-TBI study.^22^ High-frequency signals (125 Hz) were collected from the ICU-monitoring devices through the Moberg CNS unit (Moberg Research, Inc., Ambler, PA).

Through either a radial or femoral arterial line, connected to a transducer, ABP was obtained. An intraparenchymal strain gauge probe (Codman Microsensor^®^) or external ventricular drain was used to attain ICP. MAP and ICP were manually processed by removing artifacts that lasted longer than 3 sec with a software developed in MATLAB (MATLAB 2019b; The MathWorks Inc, Natick, MA). All indices were calculated in a software developed by us in LabVIEW (LabVIEW 12.0; National Instruments, Austin, TX). Thus, all data in this local study were handled and calculated by us. Because the patients were treated without a head-up position and the reference levels of ICP and MAP were the same, no corrections for the signals were needed.

### Signal processing

PRx was derived from the Pearson correlation between MAP and ICP, by calculating the correlation coefficient of 30 consecutive 10-sec averages each 5 min.^23^ A 5-min period was discarded if it contained an artifact. L-PRx was comprised of the same variables as PRx, but was instead derived from 20 consecutive 1-min averages every 20 min.^9^ A 1-min average was discarded if it contained an artifact. No correlation coefficient was calculated for those 20 min if the duration contained less than ten 1-min averages. PR was derived from the linear regression coefficient between a maximum of 12 consecutive 5-min averages of MAP and ICP each hour.^4^ A 5-min period was discarded if it contained an artifact. For the indices, patients were considered pressure passive or active if the mean value was higher or lower than a set threshold.^4^ The thresholds used for this study are described below. For all indices, the averages over the total monitoring time were calculated. No clinical interventions were based on either of the index values during the study.

Clinical outcome was evaluated at 6 and 12 months after injury in accordance with the Glasgow Outcome Scale-Extended (GOSE).^24^ The outcome was also dichotomized into favorable (GOSE 5–8) and unfavorable (GOSE 1–4) and dead/alive. In 1 subject, there was a missing GOSE score at 6 months' follow-up. For this patient, the GOSE was estimated with the knowledge of the GOSE at 3 and 12 months.

### Fixed thresholds for outcome prediction

Fixed index thresholds that correlate with unfavorable outcome have been proposed. For PRx, three of them are >0, >0.25, and >0.35^25,26^ and for L-PRx, >0.2.^9^ Patients with PR >0.13 were considered pressure passive, which is associated with an unfavorable outcome.^4^ The purposed thresholds were analyzed for each index, except for >0.35 for PRx because none of our patients had a PRx >0.35. Patients were categorized as above the specific threshold or not and tested against pupil dilation, pharmacological treatment, and outcome.

### Pharmacological treatment

Pharmacological treatment was recorded during the ICU stay. It was noted if the patients were given metoprolol (selective β_1_-adrenergic-receptor blocker), clonidine (α_2_-adrenergic-receptor agonist), or thiopental (rapid-onset short-acting barbiturate).

### Statistical analysis

For all statistical analyses, PASW Statistics (version 27.0.0.0.; IBM SPSS Statistics, IBM Corp., Armonk, NY) and Excel (version 16.49; Microsoft Corporation, Redmond, WA) were used.

For normally distributed, continuous data, Pearson's correlation was used and Spearman's rho when estimating associations in cases of categorically ranked data. For ordinal parameters, Wilcoxon's rank-sum test (Mann-Whiney U test) was used. For testing the relationship between categorical variables, the chi-square and Fisher's exact tests were applied. No correction was done for the effects of multiple testing.

To further investigate the relationship between the PR indices and outcome, univariate binary logistic regressions were performed. Here, the degree of model explanation was assessed by the Nagelkerke *R*^2^ goodness-of-fit measure. The outcome groups were the binary dependent variables, and PRx, L-PRx, and PR were the independent variables. The influence of potentially confounding variables, multi-variate logistic regressions including age, sex, Glasgow Coma Scale (GCS), pupil dilation, ICP, and CPP, as well as the three indices one at a time were applied. Level of significance was set to *p* < 0.05.

## Results

### Patient characteristics

Cohort characteristics are presented in Table 1. Median age was 56 years, GCS score was 7, and the Injury Severity Score (ISS)^27^ was 34. The ISS corresponds to a severe condition.^27^ Mean ICU monitoring time was 135.7 h (±28.95, standard deviation [SD]). Total time monitored, all patients combined, was 4000 h and of these 3902 h (97.6 %) were valid for index calculations. Eight of the 29 patients had pupil dilation (defined as pupils of uneven size) when assessed in the emergency room. Data about pupil dilation were missing on 2 patients.

**Table 1.** Patient Characteristics (*n* = 29)

Median (min-max)		
	Age (years)	56 (20–80)
	GCS	7 (3–15)
	ISS	34 (20–66)
	6-month GOSE	4 (1–8)
	12-month GOSE	5 (1–8)
Mean (±SD)		
	Time analyzed (h)/subject	135,7 (±28,95)
	ICP (mm Hg)	14.60 (±4.21)
	CPP (mm Hg)	64.79 (±5.50)
	PRx	–0.044 (±0.114)
	L-PRx	0.020 (±0.135)
	PR	0.056 (±0.078)
6-month mortality, *n* (%)		4 (14)
12-month mortality, *n* (%)		5 (17)
Male, *n* (%)		22 (76)
Female, *n* (%)		7 (24)
Unfavorable outcome 6 months, *n* (%)		15 (52)
Unfavorable outcome 12 months, *n* (%)		13 (45)
Pupil dilation, *n* (%)		8 (28)

SD, standard deviation; GCS, Glasgow Coma Scale; ISS, Injury Severity Score; GOSE, Glasgow Outcome Scale-Extended; ICP, intracranial pressure; CPP, cerebral perfusion pressure; PRx, pressure reactivity index; L-PRx, long PRx; PR, pressure reactivity.

At 6 months post-injury, median GOSE was 4 (1–8) and at 12 months was 5 (1–8). At the 6-month follow-up, 15 patients had an unfavorable outcome and 4 were dead. At 12 months, 13 patients had an unfavorable outcome and 5 were dead.

### Pressure reactivity index, long pressure reactivity index, and pressure reactivity

**FIG. 1.**
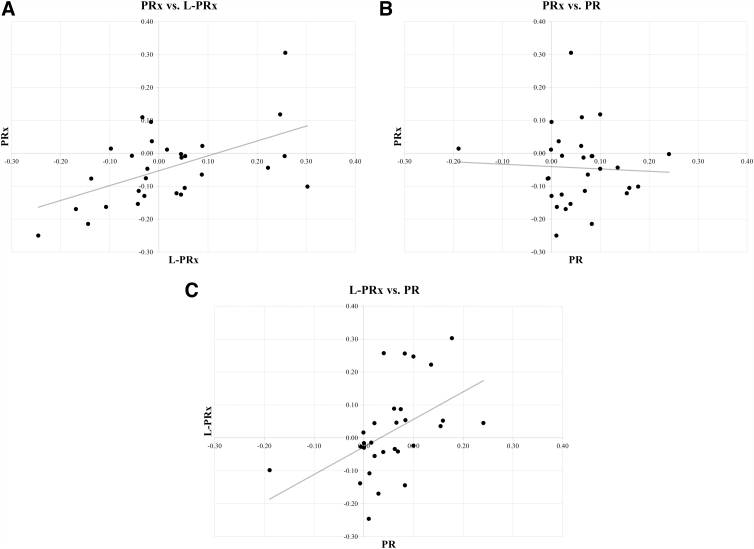
(**A–C**) Scatter plot, and Pearson correlation, of the indices plotted against each other (*n* = 29). PRx, pressure reactivity index; L-PRx, long-PRx; PR, pressure reactivity. P was < 0.01 for PRx vs L-PRx and for L-PRx vs. PR.

Correlation analyses between mean index values are presented in Figure 1A–C. PRx and L-PRx had the strongest correlation (*R* = 0.536, *p* < 0.01), a significant correlation was also observed between L-PRx and PR (*R* = 0.475, *p* < 0.01). The correlation between PRx and PR was not significant (*R* = −0.049, *p* = 0.80).

### Indices, parameters at admittance, and outcome

When patients were dichotomized according to GOSE (favorable/unfavorable, dead/alive) and pupil dilatation, there were no differences in mean indices between any of the outcome groups or pupil dilation (Table 2). There was a positive correlation between age and mean PRx (*R* = 0.482, *p* = 0.01). Logistic regression could not show a significant association between the indices and outcome. The degree of model explanation (Nagelkerke *R*^2^) ranged between 0 and 0.15 (Table 3). When the baseline parameters sex, age, GCS, pupil dilatation, ICP, and CPP were added to the models, none of the independent variables (including the indices) were statistically significant. Thus, no receiver operating characteristic analyses were performed.

**Table 2.** Comparison of Index Levels Based on Dichotomization of Pupil Dilatation and Outcome (Mann-Whitney U Test)

		PRx		L-PRx		PR	
		Mean (±SD)	*p *value	Mean (±SD)	*p *value	Mean (±SD)	*p *value
Pupil dilation at admission	Yes	–0.02 ± 0.17	*0.63*	–0.01 ± 0.13	*0.43*	0.01 ± 0.09	*0.07*
	No	–0.06 ± 0.09		0.02 ± 0.13		0.07 ± 0.06	
Outcome 6 months	Dead	–0.05 ± 0.12	*0.80*	–0.03 ± 0.07	*0.45*	0.04 ± 0.04	*0.57*
	Alive	–0.04 ± 0.11		0.03 ± 0.14		0.06 ± 0.08	
Outcome 6 months	Unfavorable	–0.05 ± 0.14	*0.38*	–0.02 ± 0.12	*0.09*	0.05 ± 0.06	*0.12*
	Favorable	–0.03 ± 0.08		0.07 ± 0.14		0.07 ± 0.09	
Outcome 12 months	Dead	–0.05 ± 0.11	*0.73*	–0.03 ± 0.06	*0.39*	0.03 ± 0.04	*0.23*
	Alive	–0.04 ± 0.12		0.03 ± 0.15		0.06 ± 0.08	
Outcome 12 months	Unfavorable	–0.03 ± 0.14	*0.93*	–0.01 ± 0.11	*0.22*	0.05 ± 0.07	*0.31*
	Favorable	–0.05 ± 0.10		0.04 ± 0.15		0.06 ± 0.09	

**Table 3.** Parameters of Univariate Binary Logistic Regression Models Modeling Clinical Outcome as a Function of Cerebrovascular Reactivity Indices

Independent variable: cerebrovascular reactivity index	Time interval for outcome assessment (months)	Clinical outcome	χ^2^ (*p*)	Nagelkerke* R^2^*
PRx	6	Favorable/Unfavorable	0.274 (0.601)	0.013
		Dead/Alive	0.005 (0.944)	0.000
	12	Favorable/Unfavorable	0.188 (0.665)	0.009
		Dead/Alive	0.042 (0.837)	0.002
L-PRx	6	Favorable/Unfavorable	3.427 (0.064)	0.149
		Dead/Alive	0.675 (0.411)	0.042
	12	Favorable/Unfavorable	1.329 (0.249)	0.060
		Dead/Alive	0.858 (0.354)	0.048
PR	6	Favorable/Unfavorable	0.577 (0.448)	0.026
		Dead/Alive	0.220 (0.639)	0.014
	12	Favorable/Unfavorable	0.129 (0.719)	0.006
		Dead/Alive	0.678 (0.410)	0.038
				

There was a significant difference regarding pupil dilation between dead/alive at 12 months (*p* = 0.017, Fisher's exact test). Eighty percent compared to 18% of patients who were dead versus alive after 12 months had pupil dilation at admission.

When specific thresholds were applied for dichotomization of the indices, neither the proportion of patients with dilated pupils, unfavorable/favorable, or dead/alive outcome between groups were significantly different (Table 4).

**Table 4.** Comparison of the Index Means Over Threshold Based on Dichotomization of Pupil Dilatation and Outcome (Fisher's Exact Test)

		PRx >0			PRx >0.25			L-PRx >0.2			PR >0.13		
		Yes	No	*p *value	Yes	No	*p *value	Yes	No	*p *value	Yes	No	*p *value
Pupil dilation at admission	Yes	4	4	*0.15*	1	7	*0.30*	1	7	*1.0*	0	8	*0.29*
	No	3	16		0	19		3	16		4	15	
Outcome 6 months	Dead	1	3	*1.0*	0	4	*1.0*	0	4	*1.0*	0	4	*1.0*
	Alive	7	18		1	24		5	20		5	20	
Outcome 6 months	Unfavorable	3	12	*0.43*	1	14	*1.0*	2	13	*0.17*	1	14	*0.17*
	Favorable	5	9		0	14		3	11		4	10	
Outcome 12 months	Dead	1	4	*1.0*	0	5	*1.0*	0	5	*0.55*	0	5	*0.55*
	Alive	7	17		1	23		5	19		5	19	
Outcome 12 months	Unfavorable	3	10	*0.70*	1	12	*0.45*	1	12	*0.34*	1	12	*0.34*
	Favorable	5	11		0	16		4	12		4	12	

### Treatment association with pressure reactivity and outcome

Twenty-two patients received metoprolol, 26 clonidine, and 6 thiopental. Two patients were given neither metoprolol nor clonidine. Information on pharmacological treatment was missing in 1 patient because of technical problems. In the outcome group dead at 6 months, 75% had received thiopental compared to 20% in the alive group (*p* = 0.038, Fisher's exact test). In the patient group who received thiopental, the GOSE median (range) at 6 months was 3 (1–6) compared to 5 (1–8) in the group who did not receive the treatment (*p* = 0.02). At 12 months, the corresponding median GOSE was 4 (1–6) compared to 6 (1–8; *p* = 0.04). No correlation between pharmacological treatments and indices was found.

## Discussion

This study investigated CA in the form of cerebrovascular reactivity and its clinical impact on patients with TBI. The index L-PRx correlated with PRx as well as PR when our ICP-oriented treatment regime was used. However, neither of the three indices correlated with outcome. Thus, we present partial negative results as compared to previous studies. Based on our results, our null hypothesis must be rejected.

Our studied cohort consisted of 29 patients, which is in line with the size of other previous studies investigating PR indices.^9,28,29^ Because only one high-frequency ICU monitor was available, data could not be collected from more than 1 patient at a time.

Mean ICP and CPP of our cohort was comparable to that of other studies.^10,21,29–31^ Mean PRx was lower in our study when compared to the CENTER-TBI high-resolution ICU cohort, where PRx was 0.038 (23% dead and 47% unfavorable outcome at 12 months), and one other study where PRx was 0.03 (24% dead and 47% unfavorable outcome at 6 months), respectively.^10,15^ PRx as well as CPP, ICP, and mean time analyzed were, however, very close to the results of a recent multi-center pilot study also investigating CA (16% dead, 51% unfavorable outcome).^29^ L-PRx as well as the mortality were lower when compared to a previous study with mean L-PRx = 0.1 (17.2% dead, 48.2% unfavorable outcome).^9^ PR was comparable to the level in a previous severe TBI study from our research group, where PR_tot_ was 0.077 (*n* = 48),^31^ and to the PR = 0.066 of a study by another group^32^ in 58 TBI patients.

The differences noted may be attributable to differences in inclusion and exclusion criteria, management method, and the degree of injury. All patients in the present study were either severely injured (judged by GCS and ISS) or their situation deteriorated to the degree that they had to be admitted to the ICU. In the group who deteriorated (*n* = 8), GCS was 15–10 (median, 13) before deterioration and intubation. These patients were also multi-traumatized with a severe injury as indicated by an ISS of 20–66 (39 ± 17) and in need of sedation and intensive care for their injuries. It has been shown in an earlier study that the degree of injury may influence the brain's response to different types of therapies because of a possible disruption of the blood–brain barrier in more severe cases,^33^ which may explain the lack of relationship between the indices and outcome in this study.

In our study, the level of correlation between PRx and L-PRx was similar to that in previous studies where *R* was 0.85 for patients with intracranial hemorrhage and 0.7 for patients with TBI.^12,13^ Differences in inclusion and exclusion criteria, diagnosis, management method, and cohort size might affect the correlation, but the similarity in correlation levels indicates that these factors do not have a crucial influence on the agreement.

There was a moderate correlation between L-PRx and PR. The same was not true for PRx/PR that did not correlate. That the relationship between PRx/L-PRx and L-PRx/PR was stronger than that between PRx–PR is not surprising, given that the calculations of PRx and L-PRx both are based on correlation, whereas L-PRx and PR are more related regarding the time domain. Given that all indices are thought to be related to CA, a stronger association between PRx and PR was expected. The correlation between L-PRx/PR has, for the first time, been affirmed in this study.

Overall, the indices did not differ significantly between outcome groups, nor did fixed thresholds, which diverges from previous studies.^4,5,8–10,13,21,25,26,30^ This may be an indication that the Lund concept, which is anchored in basic physiological principles around cerebral microcirculation and blood volume control, decreases mortality and morbidity and therefore negates the indices' correlation with outcome. Promising results have been shown in several other studies.^32,34–36^ It has also previously been shown that patients with impaired CA do not benefit from elevated CPP^32^ and may benefit from an ICP-oriented therapy.^4,33^ To our knowledge, no clinical prospective randomized study has shown that it is possible to regulate patients' CA from the proposed indices with a positive effect on outcome. Further studies are therefore warranted. The clinical applicability of calculating mean values of the indices over the whole monitoring time can also be questioned.^37^ ICP followed the expected pattern with higher values in the unfavorable groups.

In most treatment regimens for low CPP, vasopressors are used. These may directly interfere with vascular PR. In the treatment regime in Umeå, pressor drugs are actively avoided and β-blockade and α_2_-stimulation are used to reduce the stress response.^16,38^ β-blockade has been shown to improve clinical outcome in a variety of categories of trauma and ICU patients.^39–41^ Only 2 patients in our study did not receive direct pharmacological treatment to reduce the sympathetic drive to lower stress response. Further, our treatment regime does not advocate the use of mannitol in the treatment of high ICP for other than emergency situations during transport or surgery. Hyperosmotic drugs shrink the endothelial cells of the vessel wall and may therefore interact with the normal autoregulatory response.^42^ It has also been reported that mannitol *per se* induces a cerebral vasoconstriction that may affect vasoreactivity.^1^

In contrast to what is described above, there was a higher percentage of patients with worse outcomes, as well as a lower mean GOSE overall score in the groups who received thiopental. This was excepted given that this treatment is only given if ICP is not brought under control by other means. Interestingly, the use of thiopental did not correlate with the indices, which further disconnects the indices from an unfavorable neurological outcome in our study.

Age correlated positively with PRx. This may be attributable to an effect of arteriosclerosis on cerebral vascular reactivity, which has also been shown in a previous study.^43^

## Conclusion

In our study, the ICP- and MAP-derived indices PRx/L-PRx and L-PRx/PR were moderately correlated with each other when our ICP treatment regime was applied. Age was associated with PRx. However, there was no overall correlation between any of the indices and outcome. To determine whether the indices are interchangeable as measures of cerebrovascular PR, and to further understand the implications on the indices of an ICP-oriented treatment regime in patients with TBI, a randomized control study is warranted. Based on our results, the part of our null hypothesis concerning vascular pressure autoregulation indices and their relation to clinical outcome must be rejected.

## Abbreviations Used

ABP: arterial blood pressure
BTF: Brain Trauma Foundation
CA: cerebral autoregulation
CBF: cerebral blood flow
CPP: cerebral perfusion pressure
GCS: Glasgow Coma Scale
GOSE: Glasgow Coma Scale-Extended
ICP: intracranial pressure
ICU: intensive care unit
ISS: Injury Severity Score
L-PRx: long-PRx
MAP: mean arterial pressure
PR: pressure reactivity
PRx: pressure reactivity index
SD: standard deviation
TBI: traumatic brain injury
